# Supplementary material for: RinRK1 enhances NF receptors accumulation in nanodomain-like structures at root-hair tip
Source: Nat Commun. 2024 Apr 26;15:3568. doi: 10.1038/s41467-024-47794-4 (PMC11053012; doi:10.1038/s41467-024-47794-4)
Supplement: Supplementary file 1 — Supplementary information [file 41467_2024_47794_MOESM1_ESM.pdf]

RinRK1 Enhances NF Receptors Accumulation in Nanodomain-like structures at root hair tip

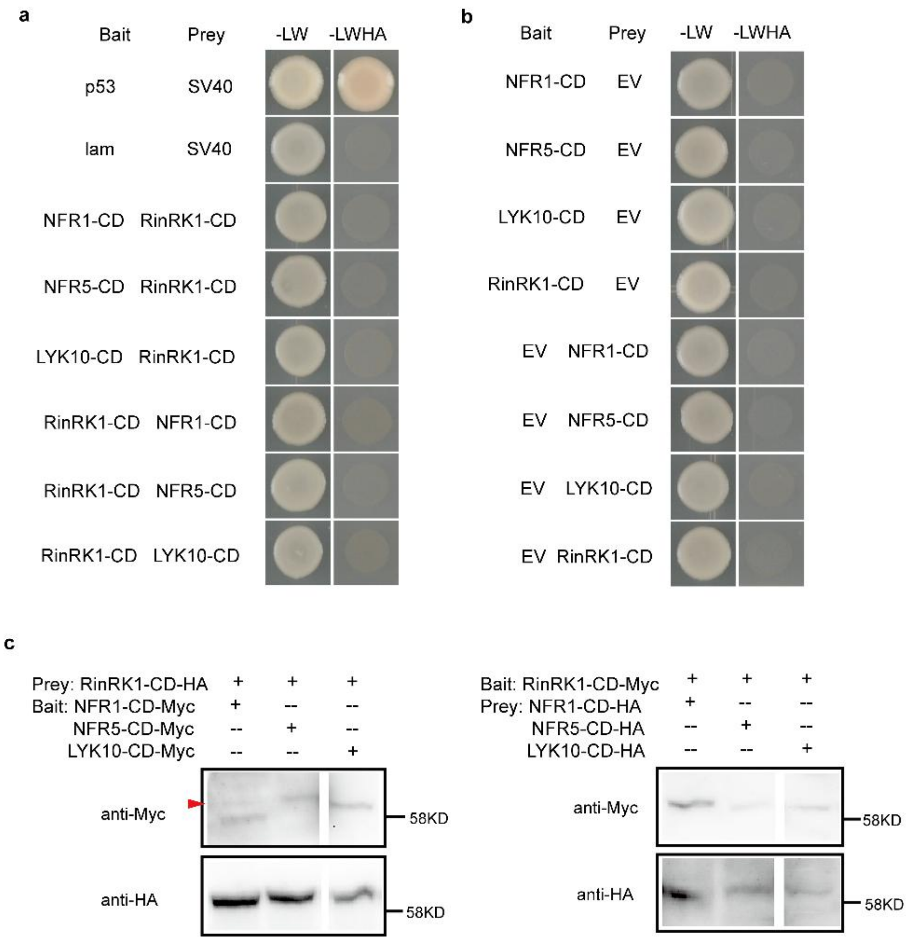

Supplementary Fig. 1 Lack of interaction between the cytoplasmic domain (CD) of RinRK1 and NFR1 or NFR5. The potential interactions between the CD of RinRK1 and NFR1, NFR5, or LYK10 were analyzed using the GAL4 yeast two-hybrid system. (**a**) The yeast cells expressing the CD of RinRK1 and NFR1, NFR5 or LYK10 did not grow on the selective medium. SV40 and p53 served as positive control, while SV40 and lam were negative control. (**b**) None of the constructs co-expressed with empty vector grew on the selective medium. (**c**) Western blot analysis for Supplementary Fig. 1a. NFR1-CD-Myc band is indicated by the red triangle.

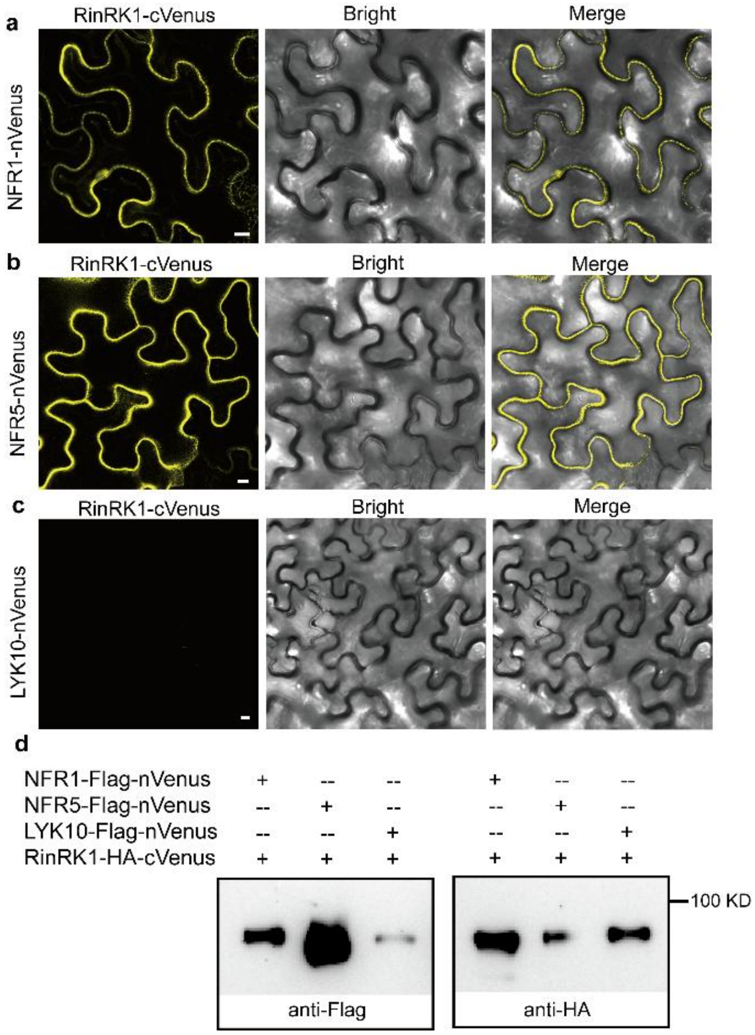

Supplementary Fig. 2 NFR1, NFR5 but not LYK10 interact with RinRK1 in the plasma membrane in *N. benthamiana* leaves. Bimolecular fluorescence complementation assay of RinRK1-cVenus-NFR1-nVenus (**a**), RinRK1-cVenus-NFR5-nVenus (**b**) or RinRK1-cVenus-LYK10-nVenus (**c**) expressed in *N. benthamiana* leaves. Bars = 10 μm. (**d**) Western blot analysis for Supplementary Fig. 2a-2c.

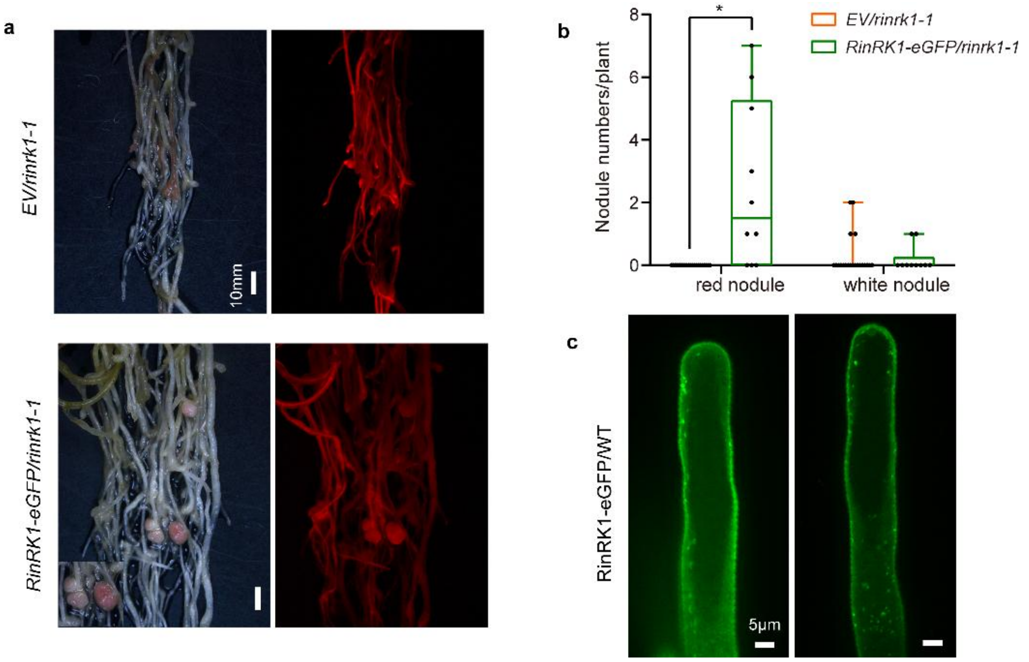

Supplementary Fig. 3 Complementation of the *rinrk1-1* nodulation phenotype by *RinRK1-eGFP*. Nodule phenotype (**a**) and number (**b**) observed in *rinrk1-1* hairy roots expressing the empty vector control (*EV*) and p*Ub:RinRK1-eGFP* at 18 dpi with *M. loti* R7A/lacZ. Bars = 10 mm. n = 21 (*EV/rinrk1-1*), n = 10 (*RinRK1-eGFP/rinrk1-1*). Asterisks indicate significant differences between the *EV* control and the experimental group (*$P < 0.05$; calculated using two-tailed Student's *t*-test). (**c**) The punctate distribution of RinRK1 in WT hairy roots. Bars = 5 µm. Boxplots show the median, upper and lower quartiles, and whiskers indicating the maximum and minimum values.

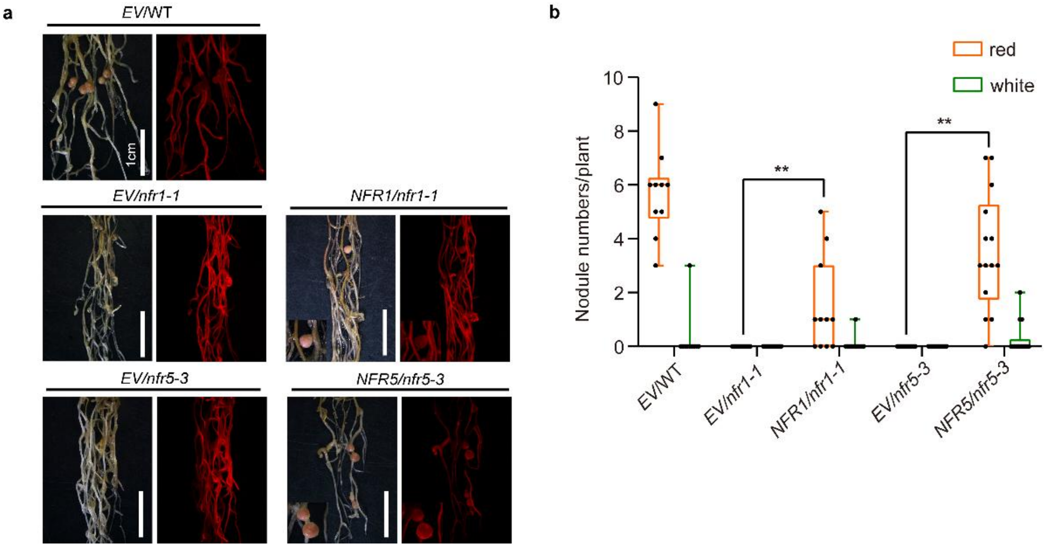

Supplementary Fig. 4 Complementation of the *nfr1-1* or *nfr5-3* nodulation phenotype by *NFR1-eGFP* or *NFR5-eGFP*. Nodule phenotype (**a**) and number (**b**) observed in *EV* control and p*35S:NFR1-eGFP* or p*35S:NFR5-eGFP* expressed in *nfr1-1* or *nfr5-3* hairy roots, and the phenotype were scored at 21 dpi with *M. loti* R7A/lacZ. Bars = 1 cm. n = 10 (*EV*/WT), n = 17 (*EV/nfr1-1*), n = 11 (*NFR1/nfr1-1*), n =14 (*EV/nfr5-3*), n = 14 (*NFR5/nfr5-3*). Statistical significance is indicated by asterisks (***P* < 0.01, **P* < 0.05; two-tailed Student's *t*-test). Boxplots show the median, upper and lower quartiles, and whiskers indicating the maximum and minimum values.

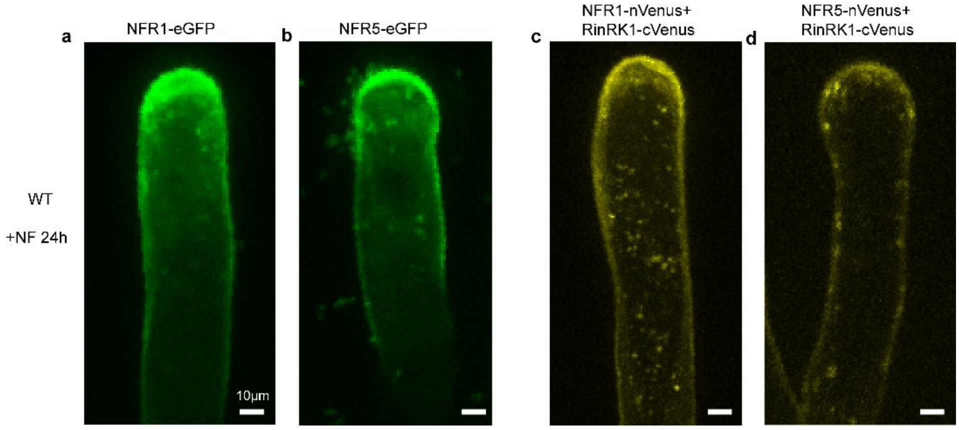

Supplementary Fig. 5 Stimulation of NFR1 and NFR5 accumulation and RinRK1 interactions by *M. loti* NFs at the root-hair tip. The punctate distribution and accumulation of NFR1-eGFP (**a**) or NFR5 (**b**) at the root-hair tip upon inoculation with *M. loti* NFs. The punctate distribution of NFR1-RinRK1 (**c**) or NFR5-RinRK1 (**d**) at the root-hair tip upon inoculation with *M. loti* NFs. Bars = 10 µm.

a

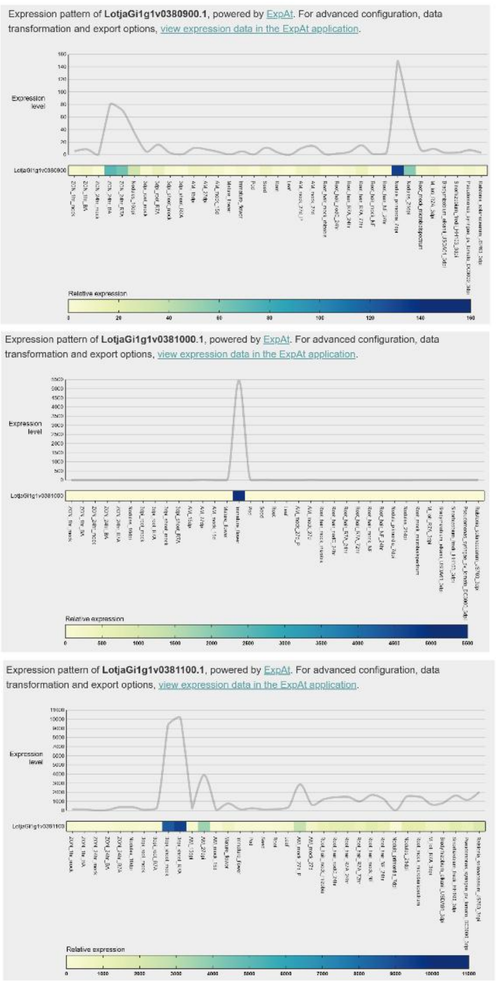

b

Gene	root-Mock	root-R7A 3dpi	root hair-Mock	root hair-R7A 24h	root hair-R7A 72h	Nodule primordia-7dpi	Nodules-10dpi	Nodules-21dpi
LotjaGi1g1v0380900	4.9	15.9	0.5	4.6	15	149.3	35.4	64.4
LotjaGi1g1v0381000	1.7	1.8	0.5	0	2.5	1.4	0.8	0
LotjaGi1g1v0381100	83.7	211.5	1242	1500.2	1001.6	35.7	370	1582

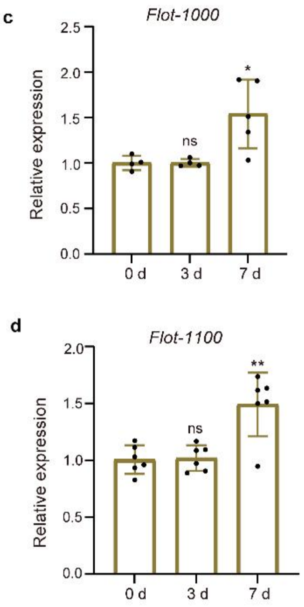

Supplementary Fig. 6 Expression patterns of *Lotus* flotillin. (**a**) Transcriptional levels of three flotillin-encoding genes in *L. japonicus*, as obtained from Lotus Base (https://lotus.au.dk/). (**b**) Quantification of the transcript levels of *flotillin* genes presented in (**a**). (**c-d**) qRT-PCR analysis of the expression levels of other *flotillin* genes in the roots of wild-type *L. japonicus* (Gifu) following inoculation with *M. loti* R7A at 0, 3, and 7 days. The expression levels were calculated relative to the corresponding expression in mock-treated samples (0 d) and normalized against the expression level of the *Lotus* ubiquitin gene. (**c**) n = 4 (0 d, 3 d), n = 5 (7 d). (**d**) n = 6. Two representative examples of three biological replicates are shown. Error bars represents mean ± sd. Statistical significance is indicated by asterisks (**$P < 0.01$, *$P < 0.05$; two-tailed Student's *t*-test).

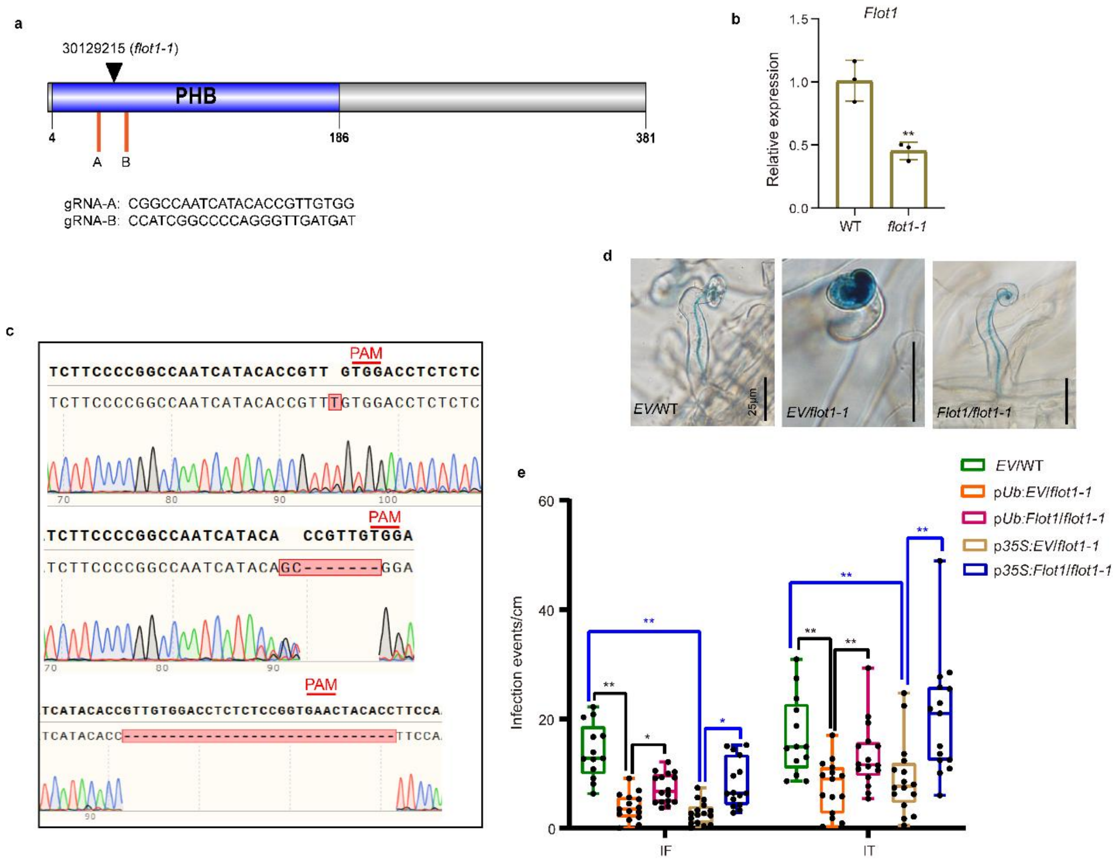

Supplementary Fig. 7 Complementation of the *flot1-1* infection phenotype by *Flot1*. (**a**) The protein structure of Flot1 is shown, indicating the insertion site of *flot1-1* and the target sites for the single guide RNA (sgRNA) in the CRISPR/Cas9-*Flot1* construct. (**b**) qRT-PCR analysis of *Flot1* transcript levels in WT and *flot1-1* roots. The expression levels were calculated relative to the expression in WT and normalized against the expression level of the *Lotus Ubiquitin*. n = 3. One representative example of three biological replicates is shown. Error bars represents mean ± sd. Asterisks indicate significant differences (***P* < 0.01; two-tailed Student's *t*-test. (**c**) The DNA sequences surrounding the designed target site for the *Flot1* locus using the CRISPR/Cas9 system. Editing types include base insertion and fragment deletion. (**d–e**) Phenotypes of infection threads or infection foci (**d**) and infection events (**e**) in wild-type or *flot1-1* hairy roots expressing the empty vector control (*EV*) and either p*Ub:Flot1* or p*35S*:*Flot1*. Normal elongating ITs in the *EV*-expressing wild-type and *Flot1*-expressing *flot1-1* hairy roots are shown. Large infection foci in the *EV*-expressing *flot1-1* hairy root is displayed. Bars = 25 µm. n = 13 (*EV*/WT), n = 15 (p*Ub*:*EV/flot1-1*), n = 16 (p*Ub*:*Flot1/flot1-1*), n = 16 (p*35S*:*EV/flot1-1*), n = 15 (p*35S*:*Flot1/flot1-1*). Significant differences are indicated by asterisks (**P* < 0.05, ***P* < 0.01; two-way ANOVA). Boxplots show the median, upper and lower quartiles, and whiskers indicating the maximum and minimum values.

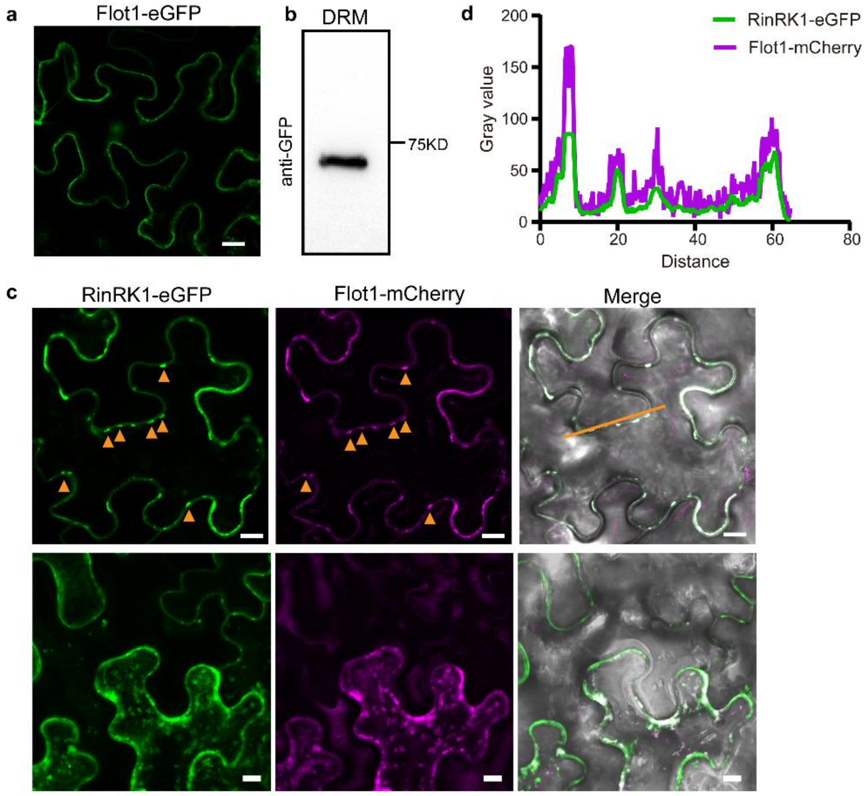

Supplementary Fig. 8 RinRK1 and Flot1 were detected in *N. benthamiana* PM nanodomain-like structures. (**a**) Confocal image of Flot1-eGFP expressed in *N. benthamiana* leaf cells, showing localization at the PM. (**b**) Immunoblot analysis using anti-GFP antibodies to detect detergent-resistant membrane proteins. Flot1 was detected in DRM. (**c**) Co-expression of RinRK1-eGFP (green) and Flot1-mCherry (magenta) in *N. benthamiana* leaves. The merged image shows punctate co-localization of RinRK1 and Flot1 in the PM. Punctate co-localization is indicated by orange triangles. The bottom images are maximum intensity z-projections of 20 optical sections. (**d**) Plots displaying fluorescence intensity analyses of RinRK1-eGFP (green) and Flot1-mCherry (magenta) using ImageJ software along the indicated lines intersecting fluorescence foci. Bars = 10 µm.

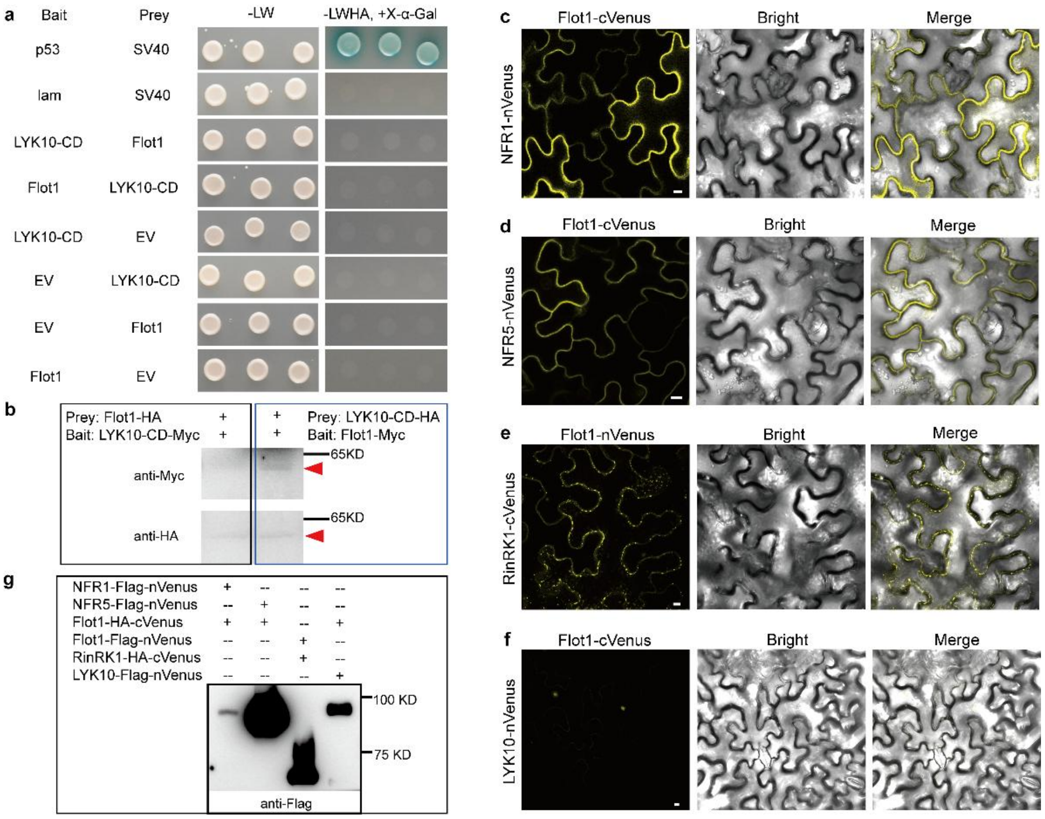

Supplementary Fig. 9 Flot1 interacts with the NFR1, NFR5, and RinRK1. (**a**) The potential interactions between Flot1 and the CD of LYK10 were analyzed using the GAL4 yeast two-hybrid system. The yeast cells expressing Flot1 and the CD of LYK10 did not grow on the selective medium. SV40 and p53 served as positive control, while SV40 and lam were negative control. (**b**) Western blot analysis for Supplementary Fig. 9 a. BiFC assays of Flot1-cVenus-NFR1-nVenus (**c**) or Flot1-cVenus-NFR5-nVenus (**d**), RinRK1-cVenus-Flot1-nVenus (**e**) or Flot1-cVenus-LYK10-nVenus (**f**) expressed in *N. benthamiana* leaves. The interactions with NFR1-Flot1 and NFR5-Flot1 mostly evenly distributed in the PM, which differed from the punctate distribution of Flot1-RinRK1. The interaction of Flot1 and LYK10 on the PM is barely observable (**f**). Bars = 10 μm. (**g**) Western blot analysis for Supplementary Fig. 9c-9f.

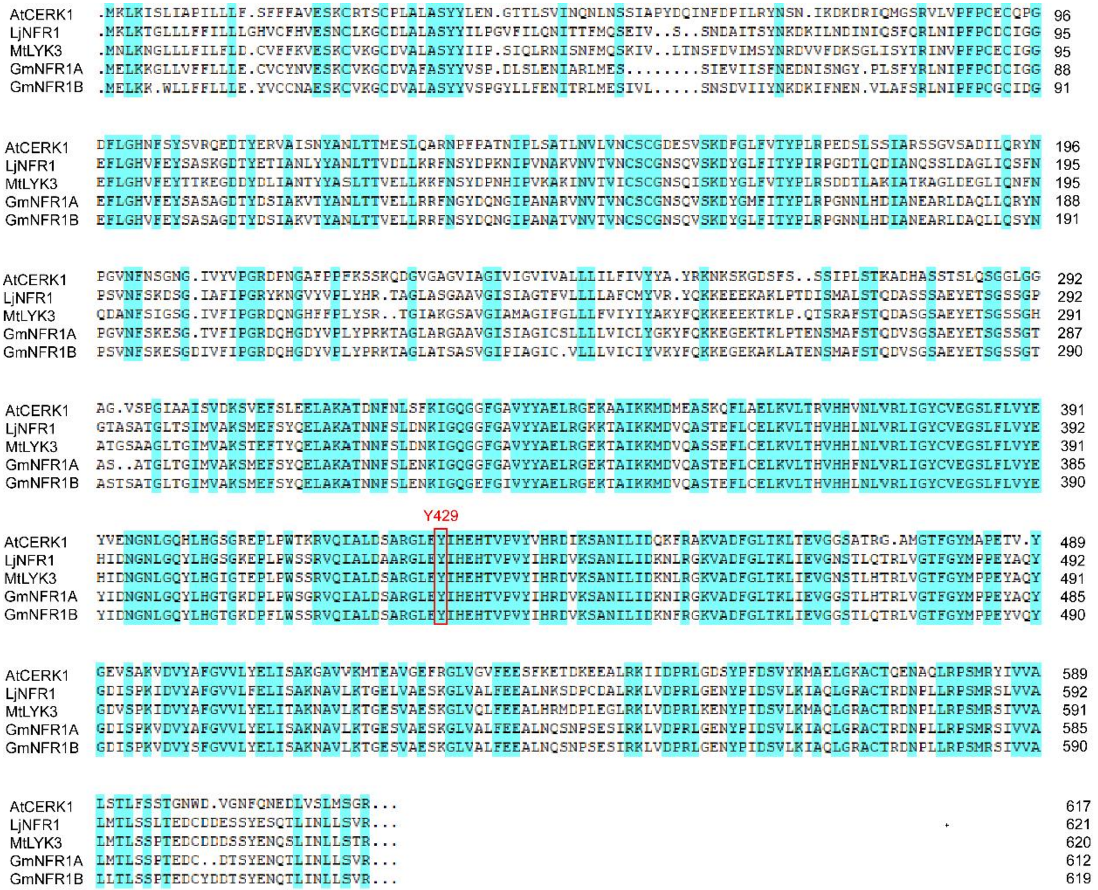

Supplementary Fig. 10 Alignment of AtCERK1 and legume NFR1 homologues. The protein sequences of AtCERK1 and legume NFR1 homologues were obtained from TAIR, Lotus Base or Phytozome. The sequences were aligned using DNAMAN 8. The alignment reveals that LjNFR1-Y429 corresponds to AtCERK1-Y428, an amino acid residue that is conserved and essential for the function of AtCERK1.

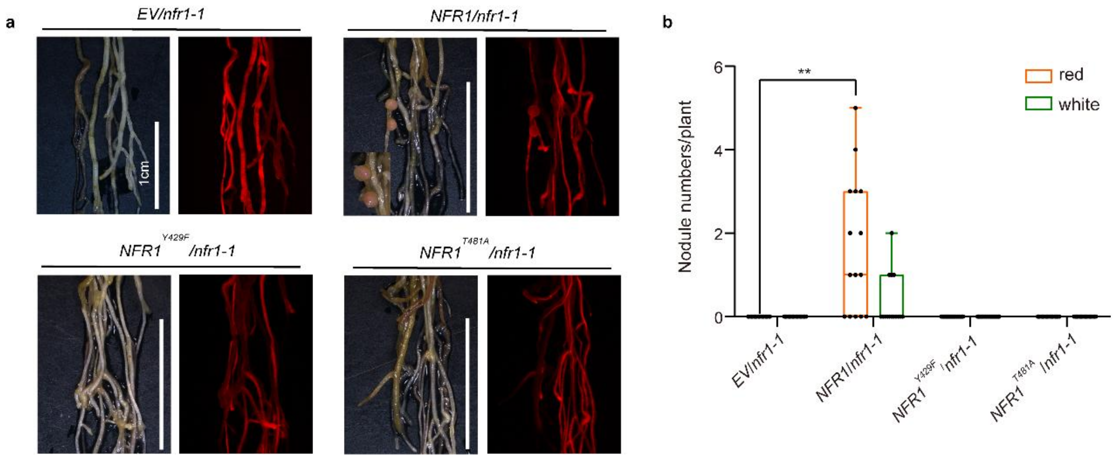

Supplementary Fig. 11 Complementation of *nfr1-1* by *NFR1^{Y429F}* and *NFR1^{T481A}*. Nodule phenotype (**a**) and number (**b**) observed in *nfr1-1* hairy roots expressing *NFR1-eGFP*, *NFR1^{Y429F}-eGFP*, *NFR1^{T481A}-eGFP*. The phenotype was scored 21 dpi with *M. loti* R7A/lacZ. Bars = 1 cm. n = 20 (*EV/nfr1-1*), n = 15 (*NFR1/nfr1-1*), n = 27 (*NFR1^{Y429F}/nfr1-1*), n = 21 (*NFR1^{T481A}/nfr1-1*). Statistical significance is indicated by asterisks (**P < 0.01; two-way ANOVA). Boxplots show the median, upper and lower quartiles, and whiskers indicating the maximum and minimum values.

Supplementary Table 1

Primer Name	Sequence
pFlot1-attB-F	GGGGACAAGTTTGTACAAAAAAGCAGGCTTCACTGGTTAACTGGCGCTCAT
pFlot1-attB-R	GGGGACCACTTTGTACAAGAAAGCTGGGTCTCTACCACAGAGTGTGTGAT
RinRK1-CCW-STE-Sfi1-F	GGCCATTACGGCCATGAGCCTAAAACCATTCTGGGCAC
RinRK1-CCW-STE-Sfi1-R	GGCCGAGGCGGCCGATGTGTCAAATGATATGGATTTTTCATC
MtLYK10-DL2xN-STE-Sfi1-F	GGCCATTACGGCCCGATGGCTTCTCTAATTCAACTTCTTTC
MtLYK10-DL2xN-STE-Sfi1-R	GGCCGAGGCGGCCGATCTTCCATCAAATACACCGCTGAAA
NFR1-attB-F	ACAAAAAAGCAGGCTTCATGAAGCTAAAAACTGGTCTAC
NFR1-attB-R	ACAAGAAAGCTGGGTCTCTCACAGACAGTAAATTTATGAG
NFR1-ED-attB-F	ACAAAAAAGCAGGCTTCAACTGTCTGAAGGGGTGTGATCTAG
NFR1-ED-attB-R	ACAAGAAAGCTGGGTCAACATACATACAAAATGCTAGTAAC
NFR1-CD-attB-F	ACAAAAAAGCAGGCTTCAGATACCAGAAGAAGGAAGAAG
NFR1-CD-attB-R	ACAAGAAAGCTGGGTCTCTCACAGACAGTAAATTTATGAG
NFR5-attB-F	ACAAAAAAGCAGGCTTCATGGCTGTCTTCTTTCTTACCTC
NFR5-attB-R	ACAAGAAAGCTGGGTCACGTGCAGTAATGGAAGTCACAATA
NFR5-ED-attB-F	ACAAAAAAGCAGGCTTCGAAAAGATTAGCGGCCCAGACTTTT
NFR5-ED-attB-R	ACAAGAAAGCTGGGTCTACGAGGGTCCCGGTTAAAACTGCA
NFR5-CD-attB-F	ACAAAAAAGCAGGCTTCTATGTATACTGCCGCAGAAAGA
NFR5-CD-attB-R	ACAAGAAAGCTGGGTCACGTGCAGTAATGGAAGTCACAATA
MtLYK10-attB-F	ACAAAAAAGCAGGCTTCATGGCTTCTCTAATTCAACTTCTTTC
MtLYK10-attB-R	ACAAGAAAGCTGGGTCTCTTCCATCAAATACACCGCTGAAA
MtLYK10-ED-attB-F	ACAAAAAAGCAGGCTTCTTACCAACTATATTTTCT

	ATTGA
MtLYK10-ED-attB-R	ACAAGAAAGCTGGGTCCACTTGTGTTTCTTTTCTT TTC
MtLYK10-CD-attB-F	ACAAAAAAGCAGGCTTCAGGAGAAATAAAGCCT ATG
MtLYK10-CD-attB-R	ACAAGAAAGCTGGGTCTCTTCCATCAAATACACC GCTGAAA
RinRK1-attB-F	ACAAAAAAGCAGGCTTCATGAGCCTAAAACCATT CTGGG
RinRK1-attB-R	ACAAGAAAGCTGGGTCTGTGTCAAATGATATGGA TTTTTC
RinRK1-ED-attB-F	ACAAAAAAGCAGGCTTCGAGGAACAAGTGGTGG TGAAGG
RinRK1-ED-attB-R	ACAAGAAAGCTGGGTCATCCTCACTGAATGATTT CTTG
RinRK1-CD-attB-F	ACAAAAAAGCAGGCTTCAAATGTATGAGGAAAT GCCAG
RinRK1-CD-attB-R	ACAAGAAAGCTGGGTCTGTGTCAAATGATATGGA TTTTTC
Kpn1-RinRK1-F	AGAGAACACGGGGGACGAGCTCGGTACCATGAG CCTAAAACCATTCTGGGC
Sal1-RinRK1-R	CGCGTACGAGATCTGGTCGACGTGTCAAATGATA TGGATTTTTC
Kpn1-NFR1-F	TACGCGTCCCGGGGCGGTACCATGAAGCTAAAAA CTGGTCTACTTT
Pst1-NFR1-R	ATACGAACGAAAGCTCTGCAGTCTCACAGACAGT AAATTTATGAGAG
Kpn1-NFR5-F	TACGCGTCCCGGGGCGGTACCATGGCTGTCTTCTT TCTTACCTCTG
Pst1-NFR5-R	ATACGAACGAAAGCTCTGCAGACGTGCAGTAATG GAAGTCACAATA
Kpn1-LYK10-F	TACGCGTCCCGGGGCGGTACCATGGCTTCTCTAA TTCAACTTCTTTC
Pst1-LYK10-R	ATACGAACGAAAGCTCTGCAGTCTTCCATCAAAT ACACCGCTGAAA
Flot1-attB-F	GGGGACAAGTTTGTACAAAAAAGCAGGCTTCATG TTGTGGAAGGTAGCAGA
Flot1-attB-R	GGGGACCACTTTGTACAAGAAAGCTGGGTCAGTG ACTTCAAATATGCTCC
RT-Flot1-F1	TTTCAATAAAGAAAAAGATCGATTATG
RT-Flot1-R1	CCACAGAGTGTGTGATTATATGTGA
RT-Flot1-F2	AGCTTAAGGCTGACTTCCTC
RT-Flot1-R2	CTAGATCTGCTTCTGCTGCT

RT-Flot (1000)-F	TACATATCCATTGGTTCATTTATTTC
RT-Flot (1000)-R	TGTTCCTTCTTTTTCCCCAAA
RT-Flot (1100)-F	CCAAGGTCCAGTCCTTGAAA
RT-Flot (1100)-R	ATAGGCACAAGGGAAGAGCA
flot1-1-F	CCCTCGATGACACCCTGGACAAGC
flot1-1-R	TCCTTCTCCACCTCTTTCCACCTCA
Flot1-T1-F	TGGTCTCGATTGCGGCCAATCATACACCGTTGGTT TTAGAGCTAGAAATAGC
Flot1-T2-R	TGGTCTCGAAACATCATCAACCCTGGGGCCGACA ATCACTACTTCGACTCTAG
Flot1-*Bbs1*-F	TTGGGTCAGAAAACTCAGATGGAGGCTGCCAATC AAGCCAA
Flot1-*Bbs1*-R	CATCTGAGTTTTCTGACCCAAGTAAGAGAAGTAC TCGTGGC
$NFR1^{Y429F}$-F	AGGCCTTGAATTCATTCATGAGCACACTGTGCCT GTGTATA
$NFR1^{Y429F}$-R	TCATGAATGAATTCAAGGCCTCTTGCTGCATCTAG AGCTAT
$NFR1^{T481A}$-F	GTCTGGTGGGAGCATTTGGATACATGCCCCCAGA ATATGCT
$NFR1^{T481A}$-R	ATCCAAATGCTCCCACCAGACGAGTTTGTAGTGT GGAGTTC
